# Research on the invulnerability and optimization of the technical cooperation innovation network based on the patent perspective—A case study of new energy vehicles

**DOI:** 10.1371/journal.pone.0238541

**Published:** 2020-09-03

**Authors:** Xia Cao, Chuanyun Li, Wei Chen, Jinqiu Li, Chaoran Lin

**Affiliations:** School of Economics and Management, Harbin Engineering University, Harbin, China; Institute for Advanced Sustainability Studies, GERMANY

## Abstract

This paper takes new energy vehicles as the research object, building the technical cooperation innovation network of new energy vehicles based on the patent perspective by establishing the related technology patent search expression, and analyzing the processes of the invulnerability and optimization in the actual technology cooperation innovation network by using the simulation analysis method. The research results show that the harmfulness of the degree value priority attack in the technical cooperation innovation network of new energy vehicles is stronger than the weighted degree value priority attack and random attack, and the attacks of the State Grid and other hub nodes have an important impact on the network invulnerability. During the network optimization process of three types of connection preferences, the “weak”-“weak” connection is the best connection mode given the situation of an unweighted network without considering the weight of the connected edge. However, the “strong”-“weak” connection is the best mode given the situation of a weighted network considering the weight of the connected edge. In addition, compared with the weighted network situation, the “strong”-“weak” connection has better network optimization results given the situation of an unweighted network. Finally, we propose counter measures and suggestions to promote the innovation network invulnerability capabilities of technical cooperation in new energy vehicles.

## 1 Introduction

The new energy vehicle industry is the "Twelfth Five-Year Plan" strategic emerging industry and one of ten key areas of industrial development "Made in China 2025". It has been an important part of the Chinese automobile industry and economic development. Technical cooperation innovation is the “energy” to promote the rapid development of the new energy vehicle industry, which represents the strategic emerging industries. Furthermore, patent cooperation is an effective method to promote the transformation of scientific and technological achievements in the process of technological cooperation innovation. In recent years, the technical cooperation innovation network based on patent cooperation has caused widespread concern in academic circles. Relevant scholars have conducted many studies on the construction, structure, evolution and operation mechanism of the technical cooperation innovation network [1–4]. However, only a few scholars have studied the invulnerability and optimization of the technical cooperation innovation network. Especially when the technological cooperation innovation network is attacked, which type of attack has a greater impact on the network invulnerability? Which nodes are more likely to cause network collapse when attacked? Which type of preferred connection mode is better for network optimization? Under the same preferred connection mode, which network type has better optimization effect? All these are issues worthy of our attention.

In terms of network invulnerability research, Albert and Barabási made pioneering research on the network invulnerability and analyzed the effect of the topology and attack strategy on network invulnerability [5]. Then, Holme studied the effect of different attack strategies on the network invulnerability and verified Albert’s conclusion [6]. Cohen transformed the network invulnerability into a percolation problem, and analyzed the invulnerability of a complex network [7]. Vázquez studied the influence of degree correlation on network invulnerability [8]. These scholars laid the foundation for the research of network survivability. On this basis, increasingly many scholars have examined the invulnerability of complex networks in the real world, such as protein networks, e-mail networks, air transport networks, and urban rail transit networks [9–12]. In the research on the invulnerability of cooperative innovation networks, some scholars use qualitative methods to study the mechanism of cooperative innovation network risk [13]. Other scholars study the stability of cooperative innovation networks based on the evolutionary game model [14]. However, Fan analysis of the impact of different attack strategies on the invulnerability of power cooperation innovation networks based on maximum flow theory [15]. Chen et al. empirically analyzed the anti-risk ability of the topology structure of the ocean energy cooperative innovation network and used the simulation analysis method to study the key factors of its anti-risk ability [16, 17]. Zhou et al. empirically analyzed the knowledge cooperative innovation network constructed by the open source product innovation community, and discussed the invulnerability of the unweighted network topology structure under different stages and different user loss patterns [18, 19]. In the research of network optimization, Shargel studied the network invulnerability optimization problem on BA model with adjustable parameters of preferred connection and network growth probability [20]. Paul et al. explored the invulnerability optimization problem of different networks with a given degree distribution [21]. Tanizawa et al. studied the network optimization problem under the simultaneous action of random attack and selective attack [22]. Wang takes the invulnerability of random attack as the network optimization objective, and transform it into the optimization problem of degree distribution entropy [23]. Beygelzimer et al. optimized the network structure from the perspective of adding edges [24]. Rezeri et al. used methods such as creating the maximum connected subgraph to optimize the network structure [25]. On this basis, some scholars have begun to study real-world network optimization problems such as urban transit networks and electric vehicle charging networks [26, 27]. In terms of optimizing the technological cooperation innovation network, Xu uses qualitative analysis methods to study the optimization path of the technological innovation network [28]. Han et al. proposed a collaborative network optimization method based on social collaboration and knowledge network, and empirically verified the effectiveness of the method [29]. Commings proposed the path to optimize the cooperative innovation network from the perspective of knowledge transfer efficiency [30]. Gu and Shao proposed to optimization strategy for optimizing the technological innovation network structure of the new energy automobile industry from three aspects of cooperation breadth, cooperation depth and cooperation efficiency [31]. In addition, Wei et al used the simulation analysis method to study the change in stability of the cooperative innovation network in three different cooperation strategies to obtain the best optimization strategy of the network [32]. The previous researches on the invulnerability and optimization of cooperative innovation network are fruitful. However, the above work still has the following shortcomings: (1) Most of the existing studies have analyzed the impact of different attack strategies on the invulnerability of the topology of the unweighted network, and lack of relevant research under the weighted network. (2) Most scholars optimize the network topology from the perspective of designing a better network, ignoring the preference connection behavior of network nodes. (3) Most of the existing researches are based on empirical analysis of real networks such as urban transportation network and air transport network, and there is a lack of lack of relevant research on the construction of technological cooperation innovation network from the perspective of patent. Thus, based on the complex network theory, this paper constructs the technical cooperation and innovation network of new energy vehicles according to the patent partnership data of new energy vehicles. On this basis, this paper from the two dimensions of network topology and network efficiency, using random attacks, degree priority attack and weighted degree priority attack to simulate the network attack to reveal the invulnerability ability of the new energy vehicle technology cooperation innovation network. In addition, the optimization process of the connection between the network nodes is simulated based on the “strong”-“strong” connections, “strong”-“weak” connections and “weak”-“weak” connections to analyze the most favorable cooperation mechanism to optimize the technical cooperation innovation network. Accordingly, this paper introduces the optimization strategy of the new energy vehicle technical cooperation innovation network in China. It has important theoretical guidance and practical reference significance for the promotion of new energy vehicle industry development and strategic development.

The remainder of this paper is organized as follows. Data sources and network construction is presented in Section 2. Then, introduction of network invulnerability and network optimization measurement methods is constructed in Section 3. Subsequently, the corresponding simulation results are given in Section 4. Finally, the conclusion and suggest are provided in Section 5.

## 2 Data sources and network construction

A patent is the product of technological innovation, which is often used as a proxy indicator of technological innovation. Patent cooperation application is widely used in studying the empirical indicators of cooperation and innovation [3, 33]. Therefore, this paper uses the new energy vehicle technology-related patents as the research subjects, which construct the network of new energy vehicle technical cooperation innovation from the patent perspective. The data source was retrieved from the patent database of National Intellectual Property Office (SIPO), and we searched for related application of new energy vehicles in the field of patents in 1989–2018. To ensure a more accurate and comprehensive collection of new energy vehicle technology-related patents, we use the high-frequency vocabulary and text-mining method [34–36] to determine the key words of new energy vehicles and the retrieval expression, which further screens new energy vehicles in the field of patent data. The specific practices are as follows.

First, we retrieve relevant literature in the field of new energy vehicles in Chinese National Knowledge Infrastructure (CNKI) and preliminary statistics related to high-frequency keywords. In addition, according to the new energy automotive industry classification of "strategic emerging industry classification" (2012) from the Chinese "Twelfth Five-Year" Plan, this paper identifies the key technologies of new energy vehicles in accordance with the high-frequency keywords divided into three technical categories: (1) battery-related technology. (2) system-related technology of the drive, gear and the working device. and (3) related technologies of charging stations and charging piles. Second, we search for patents related to high-frequency keywords according to the international IPC technology classification information to accurately screen the obtain patents. We use python software to further explore the text information related to the patent, such as names, summaries, and sovereignty items. Thus, with precise new energy vehicle technology of high-frequency keywords, we repeat the process until the total amount of patent and high-frequency keywords have small changes in the search before and after. Then, we get a more accurate and scientific new energy automobile patent search expression. Finally, through the State Intellectual Property Office (SIPO), we use a patent search platform to search for new energy-vehicle-technology-related patents. In this paper, we collected 17331 patent items related to the battery-related technology. 7662 items of systems related to the technology of drive, gear and working devices, and 14101 items related to the technologies of charging stations and charging piles. The analysis of all patent data is obtained from new energy vehicles. If a patent has two or more than two patent applicants, it is considered that there is a patent cooperation relationship between the patent applicant and the patent, based on which we selected 2413 patents with cooperative relationship as the research object of this paper.

**Fig 1:**
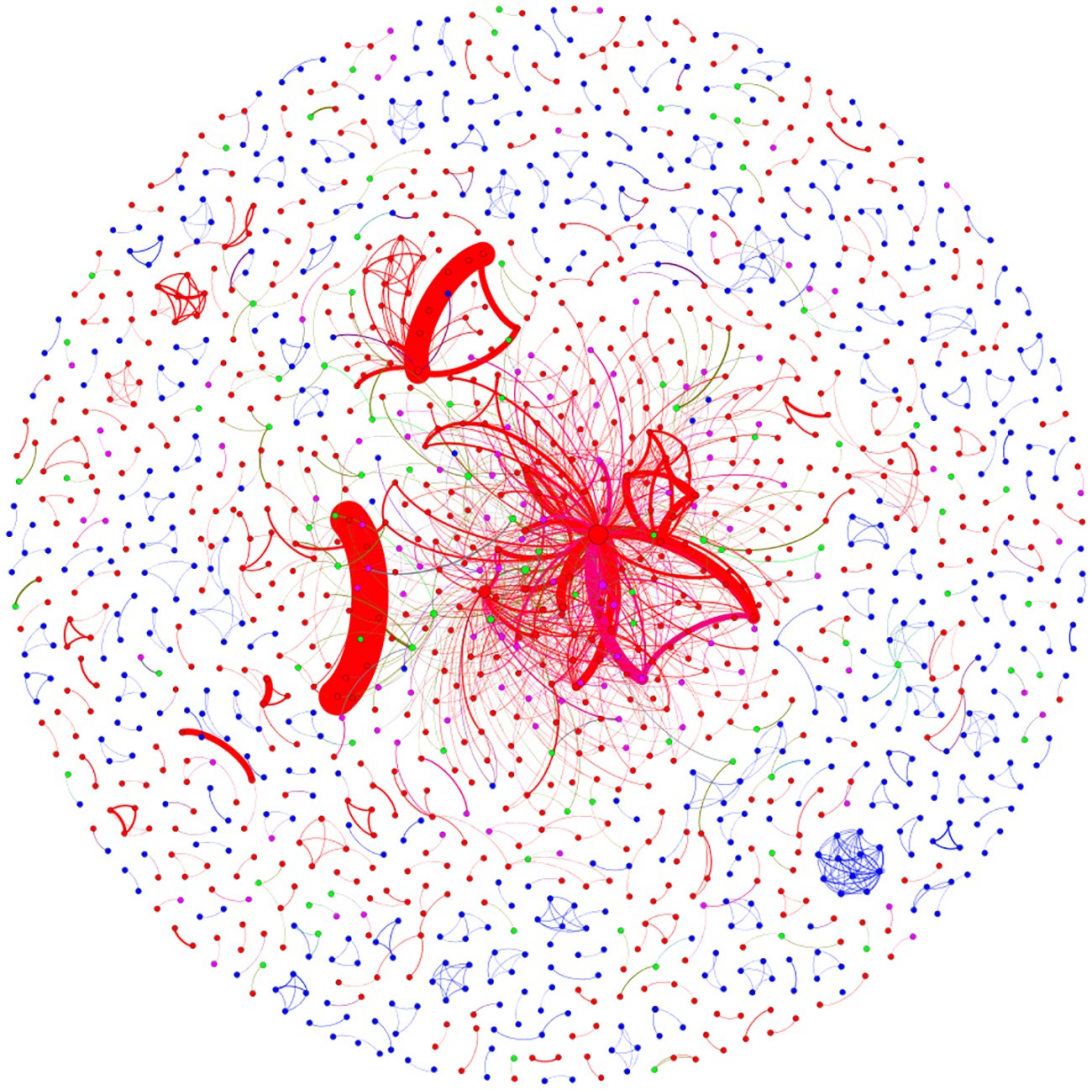
Technical cooperation innovation network map of new energy vehicles.

In this paper, 2413 patents were screened data visualization, and the new energy vehicle technology cooperation and innovation network map are depicted, as shown in Fig 1. In the network graph, there are 1472 nodes and 1881 connecting edges in total. Among them, each node represents a different patented inventor, the node size represents the degree of the node, and each edge represents an invent-cooperation relationship between both edges. The breadth of the connection represents both sides co-weights, that is, the number of patent cooperation.

To compare better and ensure networking visualization, this paper takes the connect weight new energy vehicle Patent Cooperation Network into the normalization process and makes the value of Weights between [0, 1].

As shown in Fig 1, the red, blue, green and purple nodes in the network represent enterprises, individuals, universities and scientific research institutes, respectively. Fig 1 shows that the technology cooperative innovation network of new energy vehicles exhibits a lower overall clustering effect, and most nodes are in a dispersed state. Among them, the State Grid Corporation of China is the main agglomeration center, and considerable many nodes are clustered through hierarchical connection. However, the other nodes are scattered in the form of multiple smaller connected pieces. In addition, Chongqing Chang’an Automobile Co., Ltd. and Zhejiang Geely Holding Group Co., Ltd. have high cooperation intensity. In the new energy vehicle technology cooperative innovation network, only the State Grid Corporation of China has become the core position node of the network, while other enterprises, universities and research institutes can gather the formation of the central node. Thus, in the new energy vehicles technology cooperative innovation network, the influence and control of enterprises, universities and research institutes are weak.

## 3 Research method

Network invulnerability refers to the ability of a network node (or edge) to complete its critical tasks in time when a random failure occurs or is maliciously attacked, and it is also defined as the ability to maintain its function [37, 38]. The network invulnerability index mainly measures the functional level change of the network system after the nodes or edges are attacked, which usually shows the global or local invulnerability of the network structure, that is, the reliability of the network topology [39]. At present, scholars often use parameters such as the maximum connected subgraph size and network efficiency to measure the network invulnerability [5, 6, 37]. Therefore, this paper measures the invulnerability of the network through four indicators: the maximum connected subgraph size, its descent rate, the network performance and descent rate. The maximum connected subgraph size *S* represents the total number of nodes in the maximum connected subgraph in the network, which reflects the overall connectivity of the network [5].

The maximum connected subgraph size descent rate Δ*S* represents the change in the overall network connectivity after the network node is attacked:

$$f(S)=\frac{\Delta S}{S}$$ (1)

where *S* is the maximum connected subgraph size. Δ*S* is the maximum change connected subgraph size before and after each attack. If *f*(*S*) is greater, the maximum connected subgraph size will decrease more, and the network connectivity is more damaged by attacks.

Network performance index *E* is the average of the reciprocal of the shortest distance between any two nodes in the network [6], which reflects the efficiency of information transmission between nodes in the network, and can also be understood as the level of cooperation between nodes:

$$E=\frac{2}{N(N-1)}\sum_{i\neq j\in G}\frac{1}{d_{ij}}$$ (2)

where *N* is the total number of network nodes, *d*_*ij*_ is the shortest distance between node *i* and node *j*.

Similar to the maximum connected subgraph size descent rate, network performance descent rate *f*(*E*) is used to measure the extent of damage to the network:

$$f(E)=\frac{\Delta E}{E}$$ (3)

where Δ*E* is the changes in network performance under attack. If *f*_*E*_ is greater, which indicates the greater decrease in network performance, the cooperation ability of network nodes is more damaged by the attacks.

## 4 Invulnerability and optimization analysis of the technical cooperation innovation network

Based on the definition of invulnerability of Wu [37] and Gao et al [39], this paper holds that the invulnerability of technical cooperation innovation network is the ability to complete the key tasks even when the network system suffers from a series of problems such as attacks and fault, i.e., the reliability of network topology. The purpose of network optimization is to improve the efficiency of information transmission in the network through the cooperation mechanism with different preferences to enhance the anti-destruction ability of the network.

### 4.1 Technical cooperation innovation network invulnerability simulation

#### (1) Types of network attacks

This paper draws on previous research results on the invulnerability of complex networks [6, 40–42], two modes of random attack and targeted attack are selected to analyze the invulnerability of the new energy vehicle technical cooperation innovation network. Considering that the cooperation relationship between nodes in the network is different, this paper sets up two types of targeted attack: degree value priority attack, which only considers the degree value of nodes, and weighted degree value priority attack, which considers both degree value of nodes and weight value of the connecting edge among the nodes. Among them, the degree value reflects the number of nodes connected to the nodes in the network, and the weight value is the frequency of cooperation between nodes.

#### (2) Network invulnerability analysis

**Fig 2:**
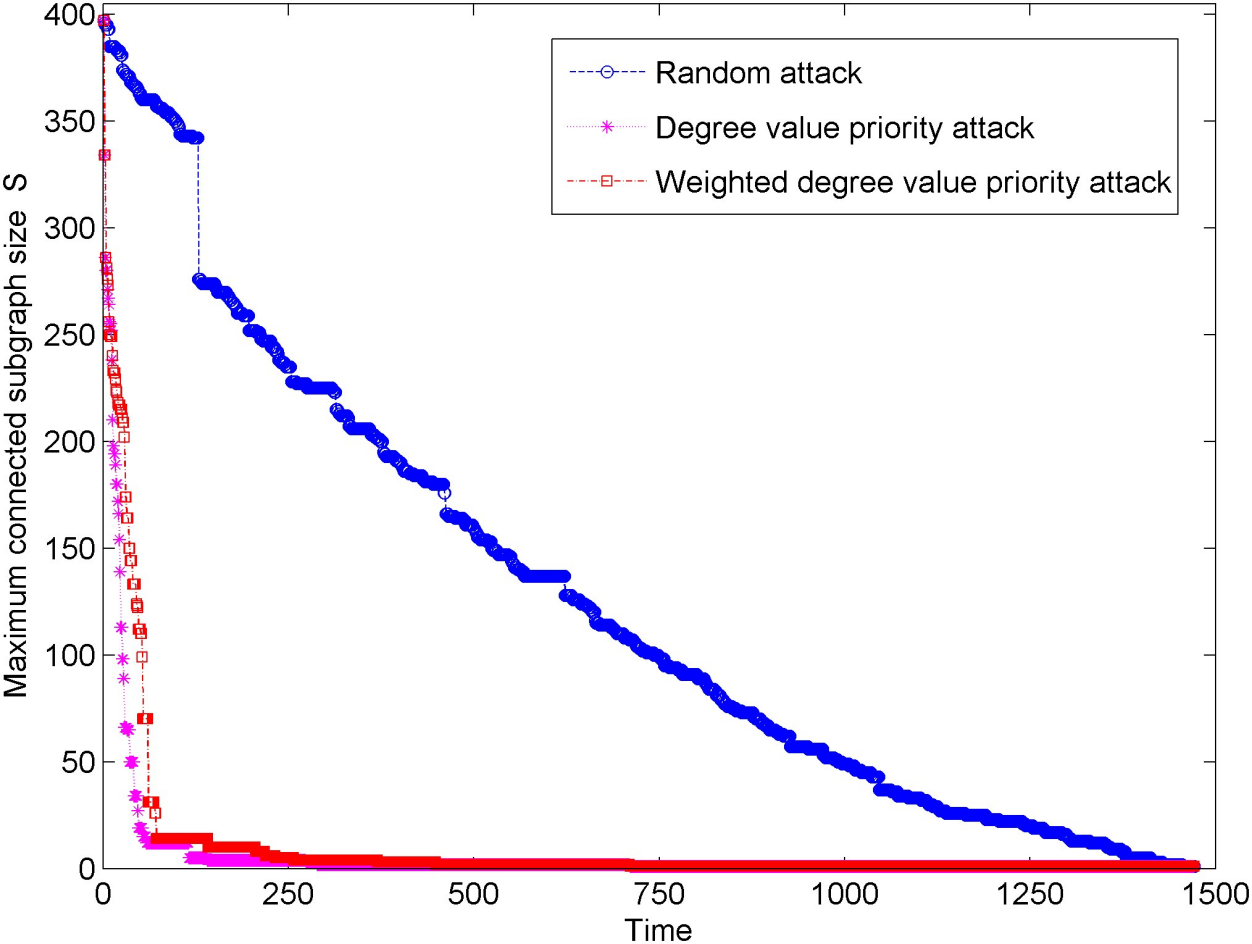
Change in maximum connected subgraph size under three types of attacks.

Fig 2 shows the change trend of the maximum connected subgraph size of new energy vehicle technical cooperative innovation network under three types of attacks. In Fig 2, when the new energy vehicle technology cooperation innovation network suffers 141 degree priority attacks and 258 weighted degree priority attacks, the change in maximum connected subgraph size tends to be stable. In the early stage of network attack, when the hub node is attacked by a degree priority attack and a weighted degree priority attack, the maximum connected subgraph size has a significant downward trend. Therefore, the hub node is the key to the invulnerability ability of the new energy vehicle technology cooperation innovation network. However, when the new energy vehicle technology cooperation innovation network suffers from random attacks, the decrease in maximum connected subgraph size is very slow, and the network is completely paralyzed until it suffers 1471 attacks. Thus, compared with the random attacks, the degree priority attacks and weighted degree priority attacks have stronger destructive power to the network. In addition, under the degree value priority attack, the maximum connected subgraph size decreases faster, which indicates that the degree value priority attack is more destructive to the network than the weighted degree priority attacks.

**Fig 3:**
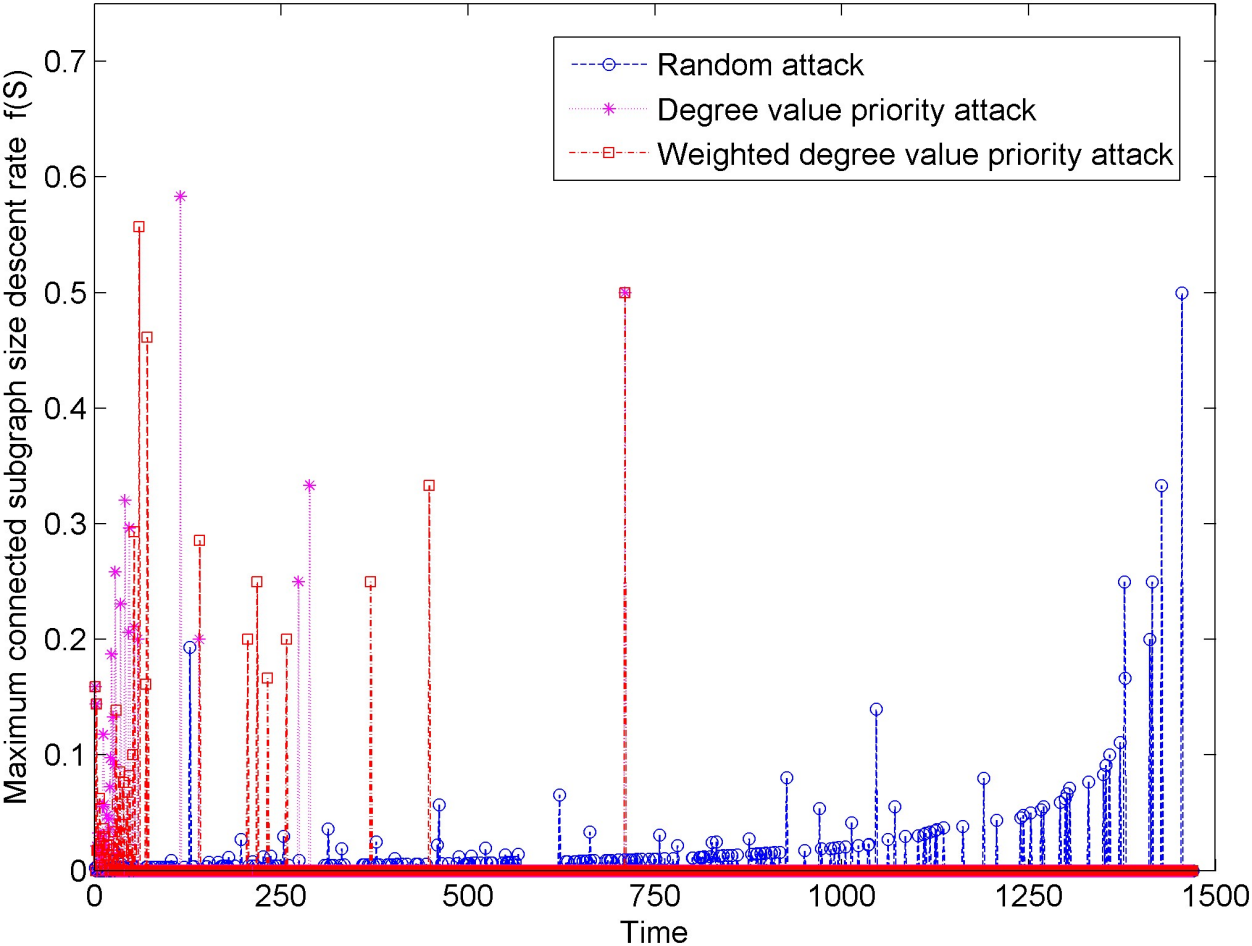
Change in maximum connected subgraph size descent rate under three types of attacks.

Fig 3 shows the changes of the decrease in maximum connected subgraph size of the new energy vehicle technology cooperation innovation network under three types of attacks. In Fig 3, the decrease in maximum connected subgraph size under the degree priority attack is larger than that under the weighted degree priority attack. It shows that the degree priority attack has greater damage to the new energy vehicle technology cooperation innovation network. In addition, when the network suffers the first 600 random attack, the maximum connected subgraph size descent rate is relatively small, which shows that the new energy vehicle technology cooperation innovation network has a good ability to resist random attacks.

The aforementioned maximum connected subgraph size change and maximum connected subgraph size decrease rate are analyzed from two aspects: damage degree and damage intensity of the network attack, which reflect that the network structure of the new energy vehicle technical cooperation innovation network is vulnerable to the face degree value priority attack and weighted degree values priority attack and robust against random attack. Compared with the weighted degree priority attack, the change in the maximum connected subgraph size and the change in the descent rate of the maximum connected subgraph size are more significant under the degree priority attack. In addition, when the hub node suffers from a network attack, the change in the maximum connected subgraph size and the change in the maximum connected subgraph size descent rate are more significant, indicating that hub node greatly affects the invulnerability ability of the new energy vehicle technical cooperation innovation network structure.

**Fig 4:**
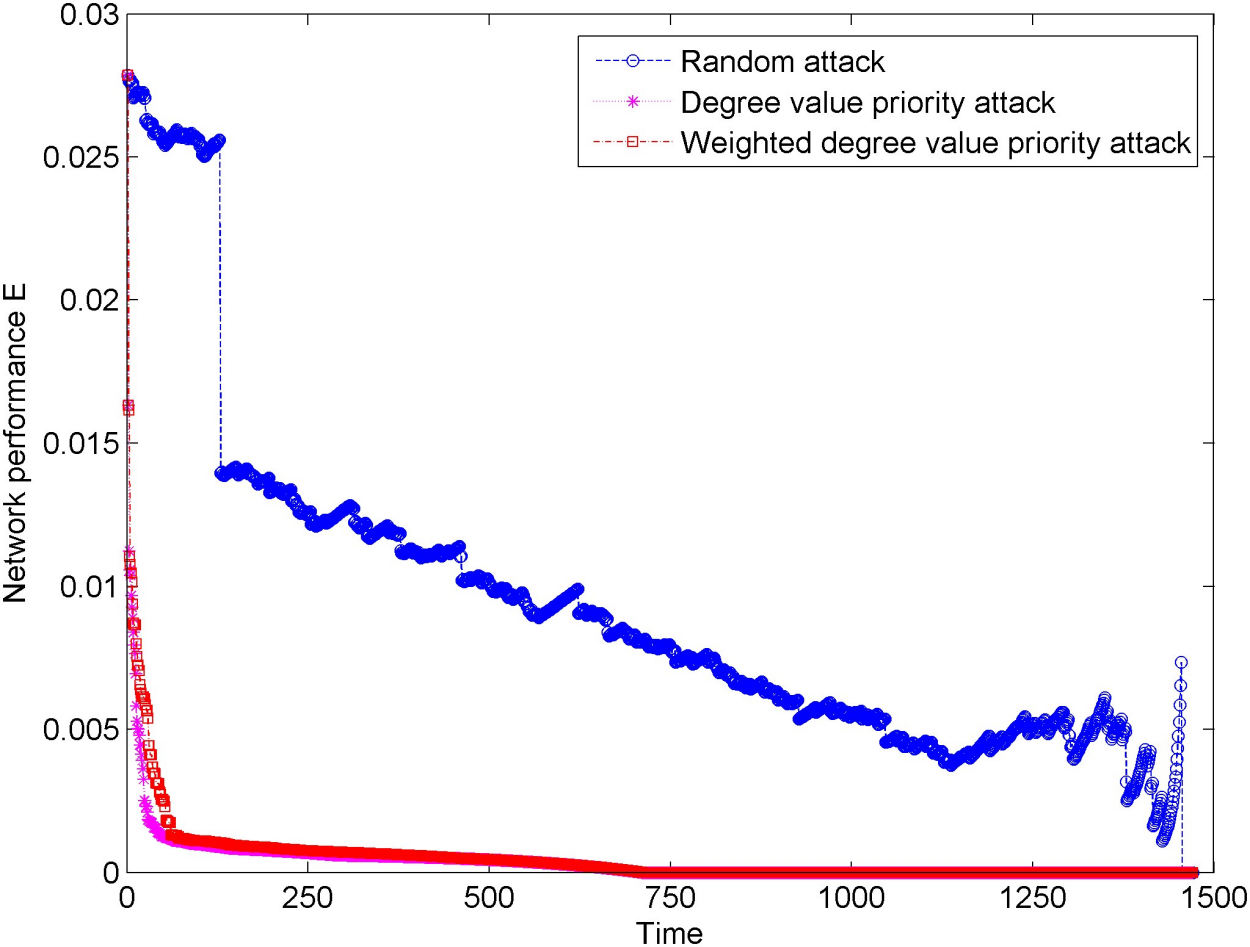
Changes of network performance under three types of attacks.

Fig 4 shows the change in network performance of the new energy vehicle technology cooperation innovation network under three types of attacks. In Fig 4, the change in network performance shows a rapid downward trend under the condition of the degree priority attacks and weighted degree priority attacks. Compared with the weighted degree priority attacks, the network performance under the degree priority attacks has a faster downward trend, and the network tends to be more quickly paralyzed. In addition, when we attack the hub node of State Grid Corporation of China, the speed of network performance decline is very obvious, which shows that the hub node in the network is the key to affect the cooperation and information transmission in the new energy vehicle technical cooperation innovation network. When the network suffers from a random attack, the overall network performance steadily changes in stages. After approximately 1100 random attacks, the change in network performance shows an increasing trend, since there are a certain number of relatively small connected pieces in addition to multiple connected giant pieces. When the network suffers a certain degree of attack and subsequent random attacks on the nodes in these smaller connected pieces, the average path length of the network decreases, so that the change in network performance will increase.

**Fig 5:**
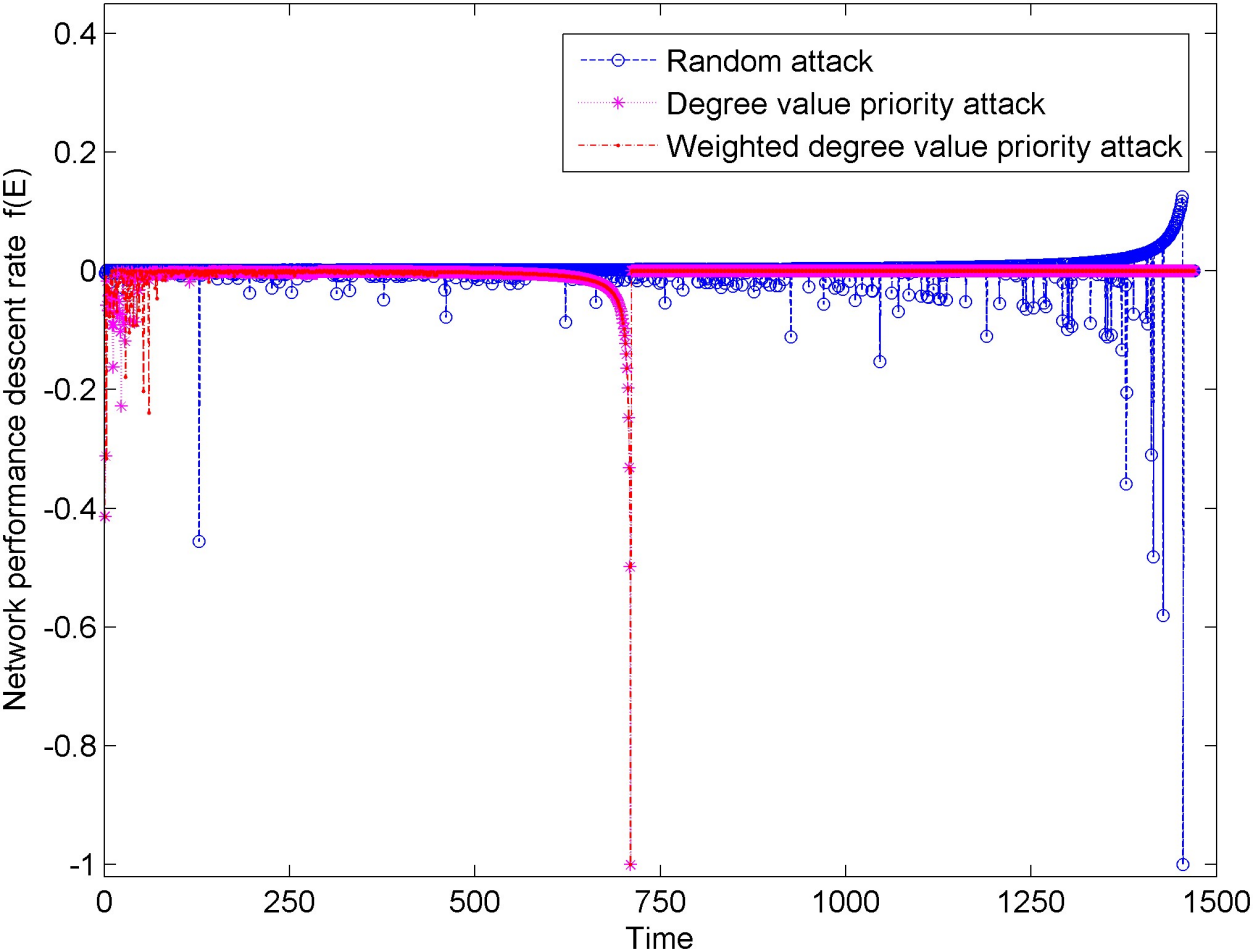
Changes of network performance descent rate under three types of attacks.

Fig 5 shows the change in network performance descent rate of the new energy vehicle technical cooperation innovation network under three types of attacks. In Fig 5, when the network suffers from the degree priority attacks and weighted degree priority attacks, the network performance descent rate has significant fluctuations, especially during the first 50 attacks. Compared with the weighted degree value priority attack, the network performance under the degree value priority attack has greater fluctuations, which indicates that the network performance is more affected by the node connection breadth of the unweighted network than the weighted network considering the edge weight. In addition, when the network suffers from random attacks, the network performance descent rate is relatively stable, and large fluctuations gradually appear after 600 attacks, which indicates that random attacks have little effect on the changes in network performance.

The aforementioned network performance and network performance decline rate change from two aspects: damage degree and damage intensity of network attack. Compared with random attacks, degree priority attacks and weighted degree priority attacks greatly affect the network performance. In addition, the hub node in the network is the key to the cooperation and information transmission in the new energy vehicle technical cooperation innovation network.

### 4.2 Simulation research in network optimization

#### (1) Type of network optimization

This paper analyzes all nodes in the new energy vehicle technology cooperation innovation network, considering the situation cooperation breadth and cooperation depth. According to the distribution of degree values of nodes, this paper assumes that in the network, if the degree value of node is greater than 5, it is considered a "strong" node. If the degree value of node is less than or equal to 5, it is considered a "weak" node. In addition, this paper selects the maximum connected subgraph size and the network performance index to measure the optimization results of the new energy vehicle technology cooperation innovation network.

In the process of optimizing the new energy vehicle technology cooperation innovation network, considering the homology and heterogeneity of the network, this paper sets three optimization modes: “strong-strong” connection, “strong-weak” connection and “weak-weak” connection. The “strong-strong” connection refers to the simultaneous selection of two different "strong" nodes to cooperate, the “strong-weak” connection refers to the simultaneous selection of two different nodes from the "strong" and "weak" nodes to cooperate, and the “weak-weak” connection refers to selecting two different "weak" nodes to cooperate. In three preferred connection modes, after the nodes are connected, the degree value and weighted degree value of the nodes must be determined again, and the "strong" and "weak" nodes are measured again to ensure the accuracy of the simulation process. In this paper, according to the setting of network optimization simulation times, the relatively stable simulation results are obtained. When the number of new energy vehicle technical cooperation innovation network simulations is 250, it can ensure that the simulation results of network optimization have an obvious overall trend and do not affect the overall effect of network optimization connection.

#### (2) Network optimization process analysis

When the new energy vehicle technology cooperation innovation network is optimized, two initial networks are set: the unweighted network, which only considers the node connection breadth, and the weighted network, which considers both node connection breadth and edge weight. Then, we study the impact of these two situation and three connection preference modes on the network optimization.

In considering the situation of unweighted network, all edge weights of the new energy vehicle technology cooperation innovation network are set to 1. In this situation, only the connection breadth of the node is considered to optimize the network.

**Fig 6:**
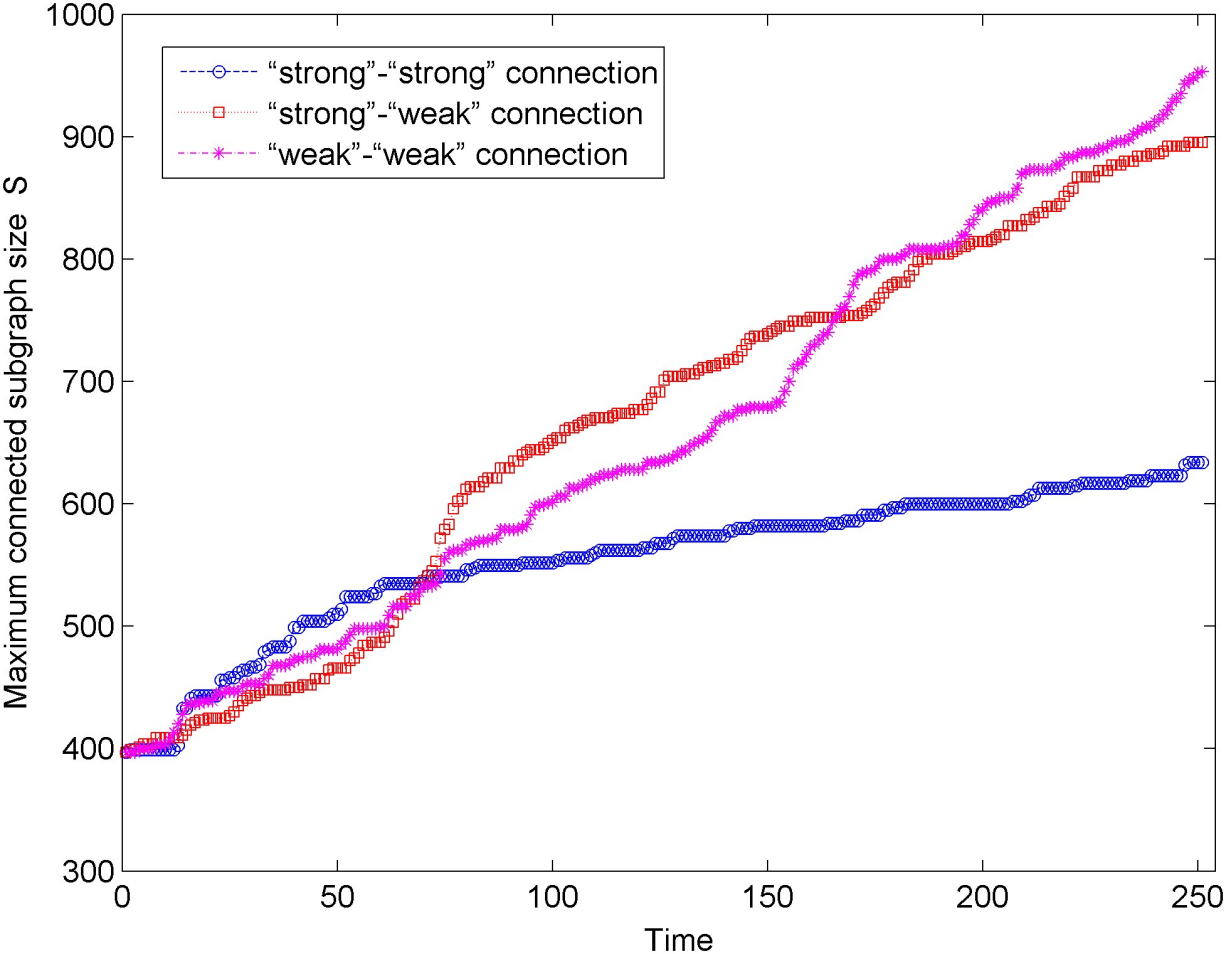
Changes of the maximum connected subgraph size in the unweighted network.

Fig 6 shows the change in maximum connected subgraph size of the new energy vehicle technology cooperation innovation network under the unweighted network situation and three connection modes. According to Fig 6, the maximum connected subgraph size of the network shows an upward trend under the three connection modes, which indicates that the network connectivity is gradually increasing. After 250 times of optimization simulation, the maximum connected subgraph size of the network changes the most in the “weak-weak” connection mode and the least in the “strong-strong” connection mode. Hence, the “weak-weak” connection is more helpful to improve the stability and connectivity of the network among the three connection modes. In addition, in the early stage of network optimization (0 < *t* < 80), compared with other connection modes, the maximum connected subgraph size has a significant increase in the “strong-weak” strong connection mode, while in the middle and late network optimization (*t* > 80), the maximum connected subgraph size more obviously changes in the “weak-weak” connection mode.

**Fig 7:**
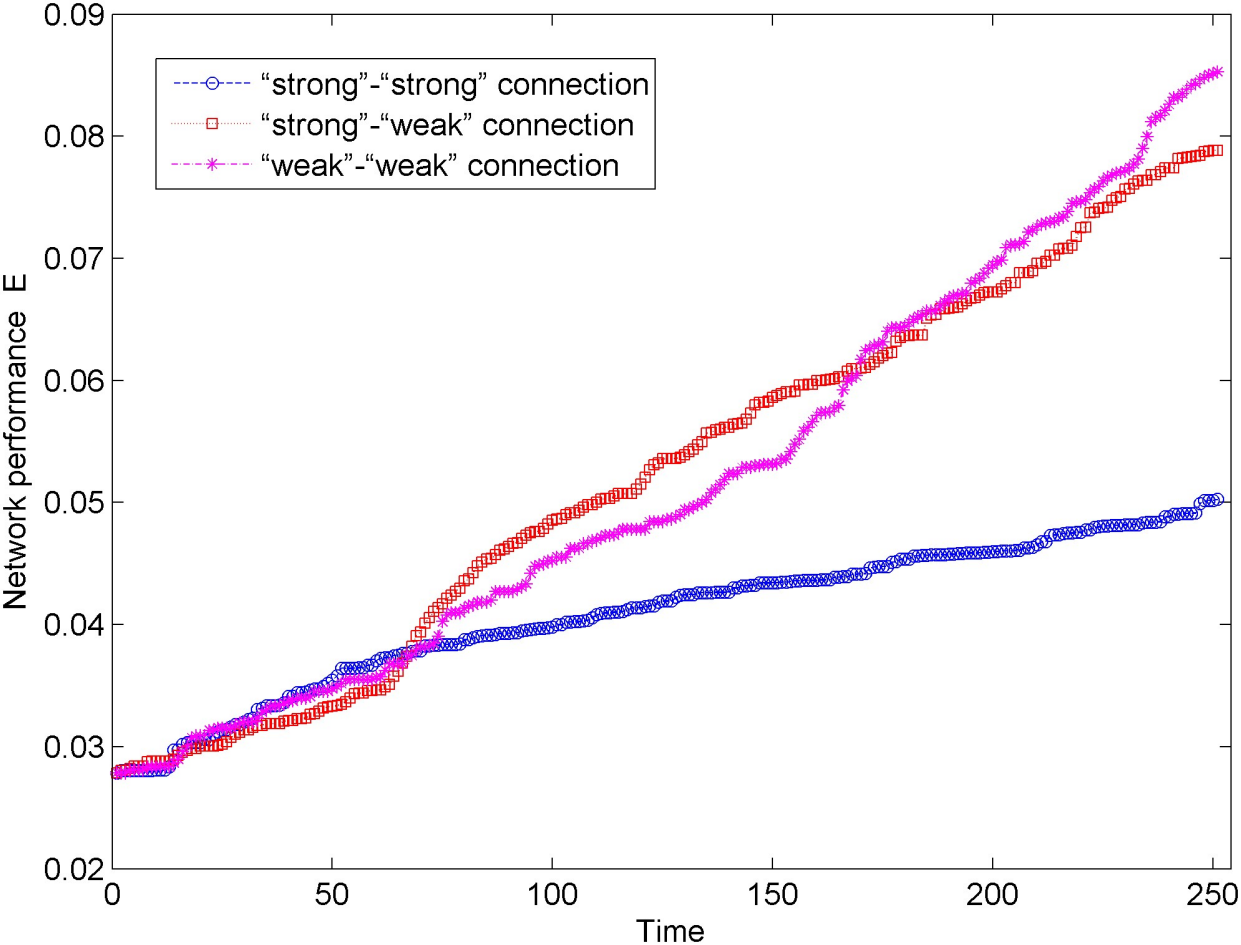
Changes of network performance in the unweighted network.

Fig 7 shows the change in network performance of the new energy vehicle technology cooperation innovation network under the unweighted network situation and three connection modes. In Fig 7, the network performance changes show a steady upward trend under the three connection modes. Compared with the “strong-strong” connection and “strong-weak” connection, the network performance has a faster upward trend under the “weak-weak” connection, which shows that the “weak-weak” connection can promote the information transmission and cooperation among the nodes in the network to improve the network invulnerability. In addition, in 250 times of network optimization, the network performance under the “strong-strong” connection mode obviously increases in the early stage of network optimization (0 < *t* < 80) but tends to be flat in the middle and late stages of network optimization (*t* > 80). The network performance under the “strong-weak” connection mode shows a relatively stable growth trend in the entire simulation process. The network performance under the “weak-weak” connection mode shows a slow growth in the early stage of network optimization (0 < *t* < 80) and a rapid growth trend in the middle and late stages of network optimization (*t* > 80).

We analyze the process of network optimization under three preferred connection modes from two aspects: the maximum connected subgraph size and the network performance. We can find that after 250 simulations, the network under the “weak-weak” connection mode has a larger maximum connected subgraph size and higher network performance, that is, the optimized network in the “weak-weak” connection mode has better invulnerability. In addition, Figs 6 and 7 show that in the early stage of network optimization (0 < *t* < 80), the maximum connected subgraph size and network performance in the “strong-strong” connection mode significantly increase. In the later stage of network optimization (*t* > 80), the maximum connected subgraph size and network performance under the “weak-weak” connection mode are more obvious.

**Fig 8:**
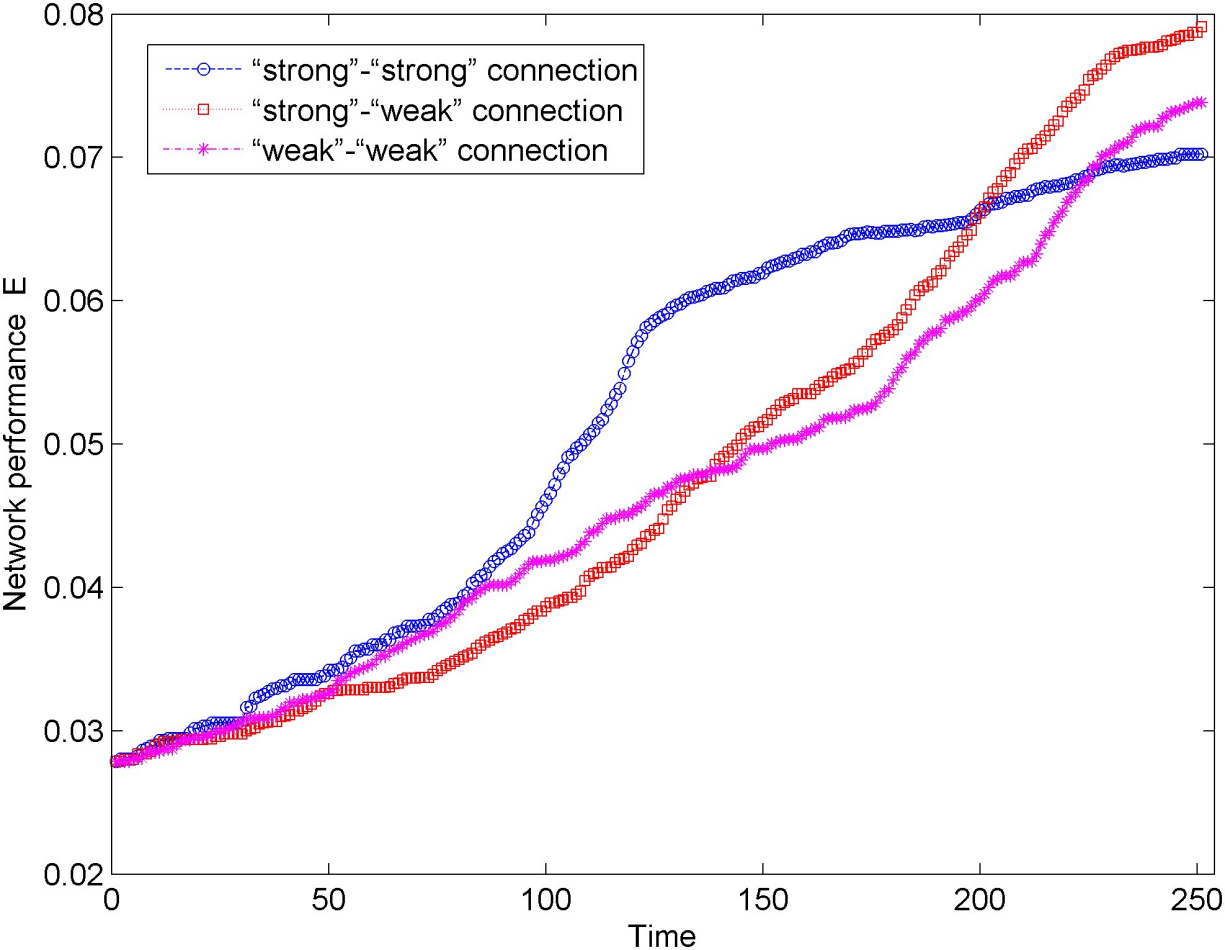
Changes of the maximum connected subgraph size in the weighted network.

In the situation of weighted network considering the connection breadth and depth of nodes, the simulation of the new energy vehicle technology cooperation innovation network is optimized. Fig 8 shows the change in maximum connected subgraph size of the new energy vehicle technology cooperation innovation network in the weighted network situation and three connection modes. In Fig 8, the maximum connected subgraph size has an obvious growth trend with three connection modes, which indicates that the network connectivity is gradually increasing. After 250 optimization simulations, the maximum connected subgraph size of the network changes the most in the “strong-weak” connection mode and the least in the “strong-strong” connection mode. Thus, among the three connection modes, the “strong-weak” connection mode is more helpful for network optimization to enhance the network invulnerability. In addition, in the 250 times of network optimization, the maximum connected subgraph size under the three connection modes shows a trend of slow growth after rapid growth. In the first and middle periods of network optimization (0 < *t* < 160), the maximum connected subgraph size in the “strong-strong” connection mode more obviously changes. In the later period of network optimization (*t* > 160), the maximum connected subgraph size in the “strong-weak” connection mode has more obvious change.

**Fig 9:**
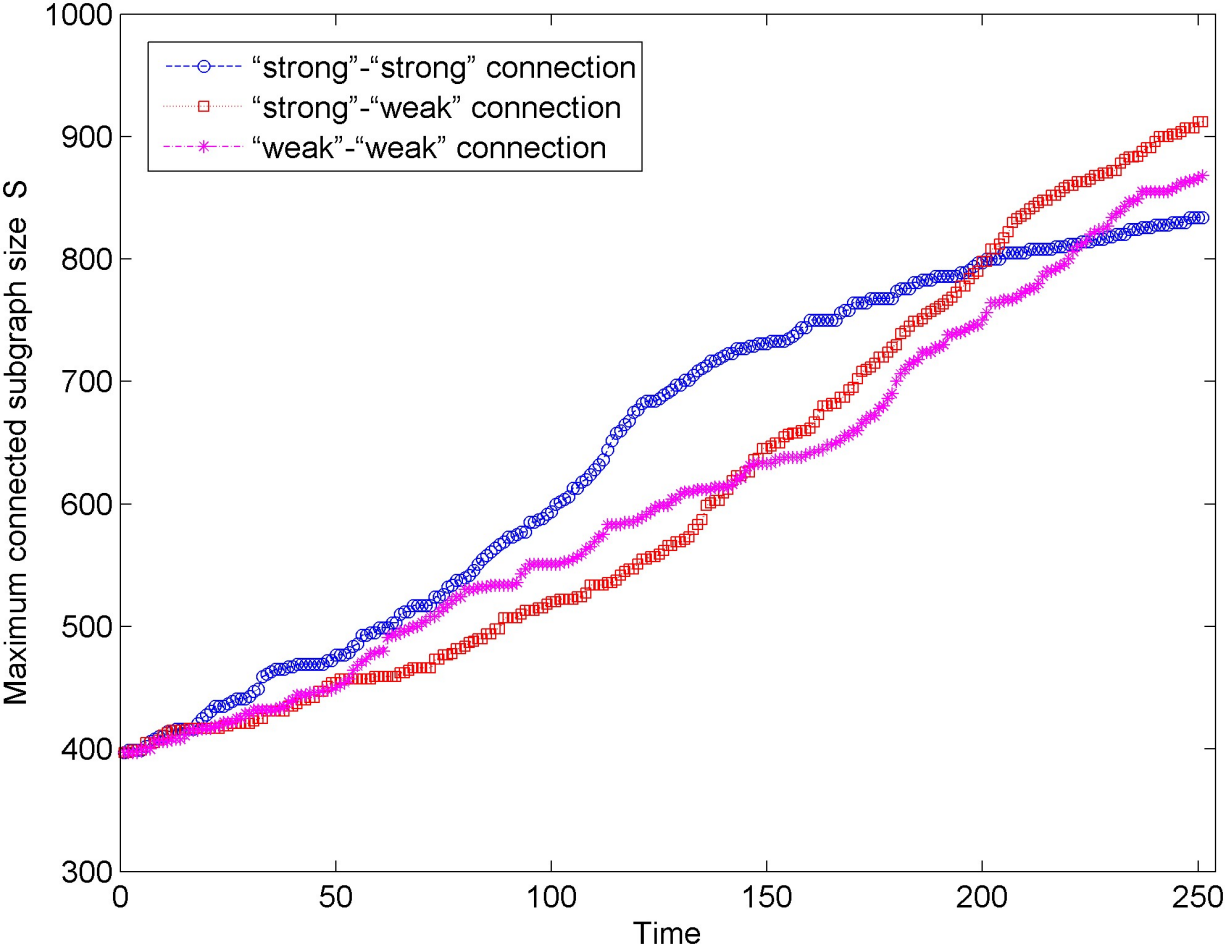
Change of the network performance in the weighted network.

Fig 9 shows the network performance changes of the new energy vehicle technology cooperation innovation network in the weighted network situation and three connection modes. According to Fig 9, we find that with three connection modes, the network performance shows a steady upward trend. After 250 optimization simulations, the network performance in the “strong-weak” connection mode changes the most, which indicates that among the three connection modes, the “strong-weak” connection mode is more helpful to promote information transmission and cooperation between nodes in the network. In addition, in the early and middle periods of network optimization (0 < *t* < 160), compared with other connection modes, the network performance has a more obvious growth in the “strong-strong” connection mode. In the late period of network optimization (*t* > 160), the network performance has a significant rapid growth in the “strong-weak” connection mode.

By analyzing the process of network optimization in the weighted network situation and three connection modes from the maximum connected subgraph size and network performance, we find that after 250 times of simulation, the network under the “strong-weak” connection mode has greater maximum connected subgraph size and higher network performance, that is, the network under the “strong-weak” connection mode has higher invulnerability. In addition, according to Figs 8 and 9, the maximum connected subgraph size and network performance under the three connection modes show a trend of rapid growth followed by a slow growth. In the early and middle periods of network optimization (0 < *t* < 160), the maximum connected subgraph size and network performance under the “strong-strong” connection mode have more obvious trends. However, in the later period of network optimization (*t* > 160), the maximum connected subgraph size and network performance under the “strong-weak” connection mode more obviously increase.

Considering three connection preference modes and comparative analysis of network optimization simulation results in two situations of unweighted network and weighted network. After 250 simulations, the optimal connection mode in the unweighted network situation is a “weak-weak” connection. The maximum connected subgraph size is 953, and the network performance is 0.0853. In contrast, the optimal connection mode in the weighted network situation is a “strong-weak” connection. The maximum connected subgraph size is 868, and the network performance is 0.0738. Therefore, compared with the weighted network, the simulation results of the network optimization in the unweighted network have a larger maximum connected subgraph size and higher network performance, so the optimized network has stronger invulnerability.

## 5 Conclusion and suggest

Based on complex network theory, according to the new energy vehicle patent cooperation relationship data, we built the new energy vehicle technical cooperation innovation network using the simulation analysis method and studied the invulnerability and network optimization process of the network from two aspects of network structure and network performance. Thus, we deeply mine the cooperation mechanism that is most conducive to the development of the cooperation and innovation network. The following conclusions and suggestions are provided.

1. In the process of new energy vehicle technology cooperation innovation network invulnerability simulation, the network has strong invulnerability to random attack, but it shows weak invulnerability to the degree value priority attack and weighted degree value priority attack. In addition, the attack of hub nodes has an important impact on the network invulnerability. According to the changes in maximum connected subgraph size and its decline rate, network performance and its decline rates, we find that the degree value priority attack is more destructive to the network than the weighted degree value priority attack. In addition, among the three attack modes, the random attack has the least damage to the network. Therefore, to promote the development of the new energy vehicle industry, it is necessary to increase the policy subsidies for enterprises engaged in the field of new energy vehicles to enhance the breadth of cooperation among the enterprises. In addition, we can promote the cooperation between universities, research institutes and enterprises to establish incubators, so it accelerates the transformation of patent achievements. In addition, the hub node composed by State Grid Corporation of China is the key to cooperation and information transmission in the network, which has an important impact on the invulnerability ability of the network structure. It is also necessary for the government to enhance the control and guidance of hub nodes, so that hub nodes can consciously strengthen cooperation with the node with smaller degrees, which promotes the knowledge interaction between the hub node and other nodes, enhances the network’s invulnerability ability and promotes the development of the new energy vehicle industry.
2. In the optimization process of the new energy vehicle technology cooperation innovation network, after 250 times of simulations, the “weak-weak” connection mode in the situation of unweighted network has the best effect on network optimization. In the situation of unweighted network, in the early stage of network optimization (0 < *t* < 80), the network optimization effect under the “strong-weak” connection is the best. In the middle and late stages of network optimization (*t* > 80), the network optimization effect under the “weak-weak” connection is the best. In the situation of weighted network, in the first and middle periods of network optimization (0 < *t* < 160), the network optimization effect under the “strong-weak” connection is better. In the later period of network optimization (*t* > 160), the network optimization effect of “strong-weak” connection is the best. Compared with the weighted network situation, the network optimization result in the unweighted network situation have a larger maximum connected subgraph size and higher network performance. Therefore, to enhance the invulnerability of the new energy vehicle technology cooperation innovation network, priority should be given to increasing the connection breadth of nodes in the network. In terms of policy, it is necessary to support the cooperative development among enterprises with smaller degree values and between enterprises with larger degrees and those with smaller degrees to form enterprise alliances and achieve complementary advantages among the enterprises. In addition, we accelerate industry-university-research cooperation to save costs, shorten the patent research cycle, and accelerate the transformation of patent achievements. Then, we can enhance the network’s invulnerability ability and promote the development of new energy vehicles.
